# 5-ALA, DTA-6, and Nitrogen Mitigate NaCl Stress by Promoting Photosynthesis and Carbon Metabolism in Rice Seedlings

**DOI:** 10.3390/metabo14030142

**Published:** 2024-02-27

**Authors:** Yaxin Wang, Chaolu Tan, Yinghao Li, Fengyan Meng, Youwei Du, Shuyu Zhang, Wenxin Jiang, Naijie Feng, Liming Zhao, Dianfeng Zheng

**Affiliations:** 1 College of Coastal Agricultural Sciences, Guangdong Ocean University, Zhanjiang 524091, China; 2112104017@stu.gdou.edu.cn (Y.W.); 202011342135@stu.gdou.edu.cn (C.T.); liyinghao@stu.gdou.edu.cn (Y.L.); 2112004001@stu.gdou.edu.cn (F.M.); zhangshuyu@stu.gdou.edu.cn (S.Z.); 2112104057@stu.gdou.edu.cn (W.J.);; 2 South China, National Saline-Tolerant Rice Technology Innovation Center, Zhanjiang 524008, China; 3 Shenzhen Research Institute, Guangdong Ocean University, Shenzhen 518071, China

**Keywords:** rice, NaCl stress, 5-ALA, DTA-6, carbon metabolism

## Abstract

A large number of dead seedlings can occur in saline soils, which seriously affects the large-scale cultivation of rice. This study investigated the effects of plant growth regulators (PGRs) and nitrogen application on seedling growth and salt tolerance (*Oryza sativa* L.), which is of great significance for agricultural production practices. A conventional rice variety, “Huang Huazhan”, was selected for this study. Non-salt stress treatments included 0% NaCl (CK treatment), CK + 0.05 g N/pot (N treatment), CK + 40 mg·L^−1^ 5-aminolevulinic acid (5-ALA) (A treatment), and CK + 30 mg·L^−1^ diethylaminoethyl acetate (DTA-6) (D treatment). Salt stress treatments included 0.3% NaCl (S treatment), N + 0.3% NaCl (NS treatment), A + 0.3% NaCl (AS treatment), and D + 0.3% NaCl (DS treatment). When 3 leaves and 1 heart emerged from the soil, plants were sprayed with DTA-6 and 5-ALA, followed by the application of 0.3% NaCl (*w*/*w*) to the soil after 24 h. Seedling morphology and photosynthetic indices, as well as carbohydrate metabolism and key enzyme activities, were determined for each treatment. Our results showed that N, A, and D treatments promoted seedling growth, photosynthesis, carbohydrate levels, and the activities of key enzymes involved in carbon metabolism when compared to the CK treatment. The A treatment had the most significant effect, with increases in aboveground dry weight and net photosynthetic rates (Pn) ranging from 17.74% to 41.02% and 3.61% to 32.60%, respectively. Stomatal limiting values (Ls) significantly decreased from 19.17% to 43.02%. Salt stress significantly inhibited seedling growth. NS, AS, and DS treatments alleviated the morphological and physiological damage of salt stress on seedlings when compared to the S treatment. The AS treatment was the most effective in improving seedling morphology, promoting photosynthesis, increasing carbohydrate levels, and key enzyme activities. After AS treatment, increases in aboveground dry weight, net photosynthetic rate, soluble sugar content, total sucrose synthase, and amylase activities were 17.50% to 50.79%, 11.39% to 98.10%, 20.20% to 80.85%, 21.21% to 33.53%, and 22.17% to 34.19%, respectively, when compared to the S treatment. In summary, foliar sprays of 5-ALA, DTA-6, and additional nitrogen fertilizer enhanced rice seedling growth, increased photosynthesis, lowered Ls values, and improved seedling salt tolerance. Spraying two regulators, 5-ALA and DTA-6, quantitatively increased the effect of nitrogen fertilizer, with comparable effects on NaCl stress regulation. This study provides the basis for efficient agricultural production.

## 1. Introduction

Rice (*Oryza sativa* L.) is one of the most important food crops worldwide. In recent years, soil salinization caused by natural changes and improper human activities has seriously threatened the growth and development of rice and even led to crop death [1,2]. More than 20% of the world’s arable land is affected by soil salinization, with irrigated areas accounting for half, which is expected to increase in the future [3]. China has 100 million hectares of saline land and 2.34 million hectares of beaches. It is expected that more than 50% of arable land will be salinized by 2050 [4]. Salt stress is the most prevalent abiotic stress globally, which hinders crop growth and development and decreases photosynthesis, mainly affecting plant morphology, physiology, and biochemistry. Salt stress inhibits the aboveground growth of plants, which ultimately reduces plant biomass [5,6]. Therefore, improving salt tolerance enhances saline land utilization and ensures global food security. Salt stress inhibits the photosynthesis and growth of rice, thereby reducing biomass and decreasing yield [7]. Therefore, improving salt tolerance in rice is an important strategy to increase rice yield.

Chemical regulation is currently one of the most effective measures to enhance NaCl stress in plants. 5-aminolevulinic acid (5-ALA) is the key precursor in the biosynthesis of all porphyrin compounds [8]. It promotes chlorophyll synthesis and facilitates photosynthesis [9]. It has been shown that foliar spraying of 5-ALA inhibits the harmful effects of different types of crop stress [10,11]. It improves plant growth and photosynthetic activity by enhancing the antioxidant system and scavenging reactive oxygen species (ROS) in tomato (*Solanum tuberosum* L.) seedlings under cold temperature stress [12]. It also enhances salt tolerance by reducing oxidative damage in Salvia miltiorrhiza Bunge (*Salvia miltiorrhiza* Bge.) [13]. Diethyl aminoethyl hexanoate (DTA-6) is a highly active plant growth regulator involved in the physiological and metabolic responses of rice crops [14]. Many studies have reported that DTA-6 improves photosynthesis and plant stress tolerance when applied as a primer, spray, seed dipping, or in combination with other plant growth regulators. For example, foliar spraying of DTA-6 improved the salt tolerance of cassia (*Catsia tora* L.) [15] and the resistance to compartmentalized stress in tomato [16] and rye (*Secale cereale* L.) [17]. It improves the photosynthesis of plants under salt stress to promote seedling growth and alleviate salt stress [18]. Nitrogen, one of the essential crop nutrients, is important for rice metabolism and yield [19]. However, excessive and improper fertilization is a serious challenge in intensive agricultural production [20,21]. Previous studies have shown that nitrogen application can alleviate salt stress. Nitrogen fertilizers mitigate the effects of salt stress on plants by improving osmoregulation and protecting cell structure and function via the accumulation of nitrogen organic compounds [22,23]. The excessive application of nitrogen fertilizers to farmland in recent years has increased environmental pollution [24]. Currently, plant regulators have been used to improve the efficient utilization of nitrogen, thereby resolving environmental challenges.

However, few comparative studies have analyzed the effects of nitrogen fertilizers and plant growth regulators on salt stress in rice seedlings. The positive effects of 5-ALA, DTA-6, and nitrogen fertilizers on plant adversity have been explored in other crops [25,26]. However, the regulatory mechanisms and beneficial effects of 5-ALA, DTA-6, and nitrogen fertilizers on salt stress in rice are still unclear. In this study, nitrogen fertilizer treatment and foliar spraying were performed on rice at the three-leaf-one-heart stage. These effects on the morphology, photosynthesis, carbon metabolites, and related enzyme activities of rice seedlings were analyzed to provide a theoretical basis for applying nitrogen fertilizer and growth regulators for the cultivation of salt-resistant rice seedlings.

## 2. Materials and Methods

### 2.1. Test Materials

The rice variety Huang Huazhan was selected for this study by the Rice Research Institute of Guangdong Academy of Agricultural Sciences (Shenzhen Longping Jingu Seed Co., Ltd., Shenzhen, China). Urea was used as the nitrogen fertilizer, 5-ALA was provided by Aladdin, and DTA-6 was provided by Zhengzhou Xinlian Biochemical Technology Co., Ltd. (Zhengzhou, China). The experiment was conducted between 2023 and 2024 in a solar greenhouse at the Guangdong Ocean University at 80 ± 5% indoor humidity and an average indoor temperature of 33 ± 2 °C during the day and 30 ± 22 °C at night.

### 2.2. Test Methods

This test was conducted using non-porous pots with an upper diameter of 20 cm, a lower diameter of 15 cm, and a height of 18 cm. The pots were cleaned and dried. Each pot was filled with 3 kg of soil (red soil: sand 3:1). The soil’s pH was 7.02. The soil’s composition was organic matter (3.52 g/kg), fast-acting phosphorus (2.62 mg/kg), fast-acting potassium (83.39 mg/kg), and alkaline dissolved nitrogen (1.26 mg/kg). The experimental treatments are listed in Table 1 and include (1) CK: 0% NaCl, (2) N: 0% NaCl + 0.05 g N, (3) A: 0% NaCl + 40 mg·L^−1^ 5-ALA, (4) D: 0% NaCl + 30 mg·L^−1^ DTA-6, (5) S: 0.3% NaCl, (6) NS: N + 0.3% NaCl, (7) AS: 40 mg·L^−1^ 5-ALA + 0.3% NaCl, and (8) DS: 30 mg·L^−1^ DTA-6 + 0.3% NaCl.

Firstly, full and intact rice seeds were selected and sterilized with 3% hydrogen peroxide for 15 min and then rinsed five times with distilled water. The sterilized and rinsed seeds were transferred to a thermostatic chamber at 30 °C in the dark for 24 h each for soaking and germination. The primer was watered before sowing. An additional 0.05 g of urea was added as N and NS treatments to randomly selected non-porous pots, which were watered with a bottom fertilizer. Seeds of consistent dewy white colors were used to dot in non-porous pots, and 69 seeds were evenly sown in each pot at a distance of 0.5 cm between seeds. Foliar sprays of 40 mg·L^−1^ 5-ALA [27] and 30 mg·L^−1^ DTA-6 [28] were applied to the three-leaf-one-center stage of seedlings under normal water management. The spray time was around 19:00 h after dark, using water as a control. The upper and lower leaves of rice were sprayed uniformly. Foliar sprays of 15 mL of 5-ALA and DTA-6 were applied to each pot. Following 24 h of conditioner treatment, the rice was treated with 0.3% NaCl [29] (*w*/*w*) based on soil mass. Salt treatments were performed by slowly pouring 9 g of salt dissolved in 1 L of water into pots to simulate the retention of a 2 cm water layer. Salinity was monitored in real-time with a soil salinometer on a daily basis to ensure appropriate salt concentration. A total of 3 replicates were conducted per treatment. Each replicate contained 10 pots of rice seedlings. There was a total of 30 pots of rice seedlings per treatment. The experiment was completely randomized, and the morphological indices of the samples were monitored on days 1, 4, 7, 10, and 13 under NaCl stress. During the sampling period, undamaged rice leaves of each treatment were cut and rapidly stored in liquid nitrogen for the determination of various physiological indices.

### 2.3. Measurement of Morphological Indices

#### Measurement of Aboveground Morphological Indicators

Samples were obtained on days 1, 4, 7, 10, and 13 under NaCl stress to measure morphological indices. Rice seedlings in each treatment were evaluated separately and rinsed with water. Fifteen representative seedlings were selected and separated above and below ground. Seedling height and leaf areas were measured with a straightedge and leaf area meter, respectively. Next, the fresh weight aboveground was measured using an electronic scale. The seedlings were heated in an oven at 105 °C for 30 min and dried at 80 °C for 48 h to a constant weight. An electronic scale was used to weigh the aboveground dry weight.

### 2.4. Measurement of Physiological and Biochemical Indicators

Triplicates were conducted for each indicator of physiology and biochemistry.

#### 2.4.1. Determination of Gas Exchange Parameters

Gas exchange parameters were determined on days 1, 4, 7, 10, and 13 of NaCl stress. The middle part of the penultimate fully expanded leaf of each seedling was evaluated between 9:00 and 11:00 a.m. on clear mornings [30]. Net photosynthetic rate (Pn), transpiration rate (Tr), stomatal conductance (Gs), and intercellular carbon dioxide (Ci) were measured using a portable photosynthesizer 6800 (LI-COR, Inc., Lincoln, NE, USA). The CO_2_ concentration in the leaf chamber was 400 μmol mol^−1^. The light intensity was set at 1000 μmol m^−2^ s^−1^. The leaf temperature was 32 ± 1 °C, and the relative humidity ranged from 70% to 80%. The following equations were used:

Apparent mesophyll conductance (AMC) = Pn/Ci

Stomatal limitation value (Ls) = (Ca − Ci)/Ca

#### 2.4.2. Histochemical Staining

Rice leaves were stained to measure superoxide anion (O_2_·^−^) and hydrogen peroxide (H_2_O_2_) levels using nitrogen blue tetrazolium (NBT) [31] and diaminobenzidine (DAB) [32] on day 7 of NaCl stress. Leaves from the same plant part in each treatment group were cut and transferred to a container of prepared reagents and vacuumed separately. The leaves were then left at room temperature in the dark for 24 h until brown and blue spots appeared. The leaves were decolorized by adding 95% ethanol to a water bath at 80 °C until they were free of chlorophyll. The leaves were then photographed.

#### 2.4.3. Determination of Osmoregulatory Substances

Soluble protein content was determined using the Thomas Brilliant Blue G-250 method described by Luo Q et al. [33]. Proline content was determined using the sulfosalicylic acid method described by Bates et al. [34].

#### 2.4.4. Determination of Ascorbic Acid (AsA) and Glutathione (GSH) Levels

Ascorbic acid (AsA) and total ascorbic acid (AsA + DHA) levels were determined using methods described by Kampfenke [35] and Costa [36] with minor modifications. The absorbance was measured at 534 nm. The DHA content was determined by subtracting the AsA level from the AsA + DHA content. Total glutathione (GSH + GSSH) and GSSG levels were determined using the dithiobenzoic acid method [37], and the absorbance was measured at 412 nm. The GSH content was determined as described by Tyburski and Tretyn [38] with minor modifications. The reaction mixture consisted of 200 μL of supernatant, 2.6 mL of acetate buffer (0.2 M, pH 5.6), and 200 μL of 5,5-disulfuric acid-(2-nitrobenzoic acid). Samples were stored at 30 °C for 10 min. The absorbance of GSH was measured at 412 nm.

Using liquid nitrogen, 0.5 g of frozen leaf samples were powdered and homogenized with 10 mL of PBS (50 mM, pH 7.8). The samples were centrifuged at 12,000× *g* for 20 min at 4 °C. The supernatant was collected as an enzyme solution extract for the determination of aseorbateperoxidase (APX), glutathione reductase (GR), dehydroascorbate reductase (DHAR), and monodehydroascorbate reductase (MDHAR) activities. APX activity was determined using a method described by Rahman [39]. The enzyme solution (0.1 mL) was treated sequentially with 0.1 mM EDTA-Na_2_, 5 mM AsA, and 20 mM H_2_O_2_, and the absorbance was measured at 290 nm. The GR activity was measured using a method described by Zhu et al. [40]. A 100-μL aliquot of the enzyme solution was treated sequentially with a 25 mM phosphate-retarded natriuretic solution (containing 2 mM EDTA, pH 7.0), 10 mM GSSG, and 24 mM NADPH. The absorbance was measured at 340 nm, and 30 s variations in absorbance were calculated. The MDHAR activity was determined using a method described by Shan et al. [41]. The supernatant was treated with a 25 mM phosphate-retarded nanoflush solution (containing 2 mM EDTA, pH 7.0), 7.5 mM AsA, 2 mM NADPH, and ASA oxidase sequentially. The absorbance values were measured at 340 nm. The DHAR activity was determined using a method described by Hasanuzzaman [42]. The enzyme solution (100 μL) was treated with a 25 mM phosphate-retarded natriuretic solution (containing 2 mM EDTA, pH 7.0), 20 mM GSH, and 10 mM DHA, and the absorbance was measured at 265 nm.

#### 2.4.5. Measurement of Indicators Related to Carbon Metabolism in Leaf Blades

Carbohydrate content was determined as described by Du et al. [43]. The frozen leaf blades (0.5 g) were first homogenized with an 80% ethanol solution (*v*/*v*) and then transferred to a centrifuge tube in a water bath at 80 °C for 20 min. The homogenate was then centrifuged for 5 min at 4000× *g* to collect the supernatant. The supernatant was extracted three times to obtain a final volume of 25 mL. The enzyme solution was used to measure sucrose, fructose, and soluble sugars. The precipitate was used to measure the starch content. The absorbance values of sucrose, fructose, soluble sugar, and starch were measured at 480 nm, 480 nm, 620 nm, and 620 nm, respectively.

Frozen leaves (0.5 g) were ground and homogenized with 0.1 M of PBS buffer (pH 7.5) containing 5 mM MgCl_2_, 1 mM EDTA, 0.1% (*v*/*v*) β-mercapto-ethanol, and 0.1% (*v*/*v*) Triton X-100. The supernatant was collected by centrifugation at 10,000× *g* and 4 °C for 15 min. The supernatant was used to measure sucrose synthase (SS), sucrose phosphate synthase (SPS), neutral invertase (NI), and acid invertase (AI) activities. SS and SPS were determined using the methods described by Baxter [44] and Wongmetha [45], and absorbance values were measured at 480 nm. NI and AI activities were determined using a method described by Zhu et al. [46]. Absorbance values were measured at 540 nm. 

α-amylase, β-amylase, and total amylase activities were determined as described by Dai et al. [47]. Then, 1 mL of DNS was added to 1 mL of the supernatant and shaken well. The sample was heated at 100 °C for 5 min and then cooled. The absorbance value was measured at 520 nm. Starch phosphate carboxylase (SP) activity was determined, as described by Singh et al. [48].

### 2.5. Data Analysis

Microsoft Excel 2010 was used for data processing. Based on the mean and standard error values obtained from the experimental data, one-way or two-way ANOVA was performed using the SPSS 25.0 software (SPSS, Inc., Chicago, IL, USA). Multiple comparisons were performed using Duncan tests. Graphs and tables were plotted using the Origin 2021 software.

## 3. Results and Discussion

### 3.1. Effects of Plant Growth Regulators (PGRs) and Nitrogen Application on Rice Seedling Morphology

**Figure 1:**
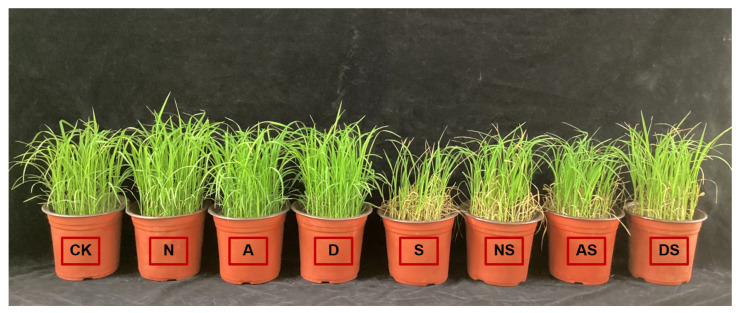
Effects of PGRs and nitrogen application on rice seedling growth (13 d). Notes: CK: 0% NaCl; N: 0% NaCl + 0.05 g N; A: 0% NaCl + 40 mg·L^−1^ 5-ALA; D: 0% NaCl + 30 mg·L^−1^ DTA-6; S: 0.3% NaCl; NS: N + 0.3% NaCl; AS: 40 mg·L^−1^ 5-ALA + 0.3% NaCl; DS: 30 mg·L^−1^ DTA-6 + 0.3% NaCl.

As shown in Figure 1 and Table 2, exposure to both salt concentration (Sc) and conditioning factor (Re) significantly affected seedling height, leaf area, aboveground fresh weight, and aboveground dry weight (*p* < 0.01). NaCl stress decreased seedling height, leaf area, aboveground fresh weight, and aboveground dry weight. The seedling height, leaf area, aboveground fresh weight, and aboveground dry weight showed a decrease of 0.12–8.58%, 13.08–25.40%, 5.07–29.58%, and 10.75–23.14%, respectively, in the S treatment when compared to the CK treatment between days 1 and 13 after NaCl stress, indicating that NaCl stress inhibited rice seedling growth. The increased application of nitrogen fertilizer promoted seedling growth. Exposure to NaCl stress increased the seedling height, leaf area, aboveground fresh weight, and aboveground dry weight in the NS treatment by 1.95–20.77%, 8.97–21.81%, 3.30–28.63%, and 9.63–30.79%, respectively, when compared to exposure to the S treatment, which indicated that the increased application of nitrogen fertilizer alleviated the toxic effects of NaCl stress on rice seedling growth. Exposure to 5-ALA after 4 to 13 days of salt stress led to an increase in seedling height, leaf area, aboveground fresh weight, and aboveground dry weight by 6.29–22.71%, 3.74–22.49%, 9.37–39.33%, and 17.74–41.02%, respectively, in the A treatment when compared to CK. The seedling height, leaf area, aboveground fresh weight, and aboveground dry weight in the AS treatment also increased by 12.61–27.59%, 10.55–19.96%, 5.01–46.23%, and 17.50–50.79%, respectively, in the AS treatment when compared to the S treatment. The DS treatment also increased seedling height, leaf area, aboveground fresh weight, and aboveground dry weight between days 4 and 13 after NaCl stress, indicating that the spraying of the two PGRs not only promoted the morphological growth of seedlings but also alleviated the damage induced by salt stress. Following 13 days of NaCl stress, the seedling height, leaf area, and aboveground dry weight increased in the AS and DS treatments when compared to the NS treatment, with significant increases of 14.78% and 7.35% in seedling height, 4.83% and 3.29% in leaf area, and 4.99% and 1.49% in aboveground dry weight, respectively. Both PGRs increased the effect of nitrogen fertilizer application in promoting rice seedling growth, particularly ameliorating the damage caused by NaCl stress.

### 3.2. Effects of 5-ALA, DTA-6, and Nitrogen Application on the Photosynthetic Characteristics of Rice Seedlings

**Figure 2:**
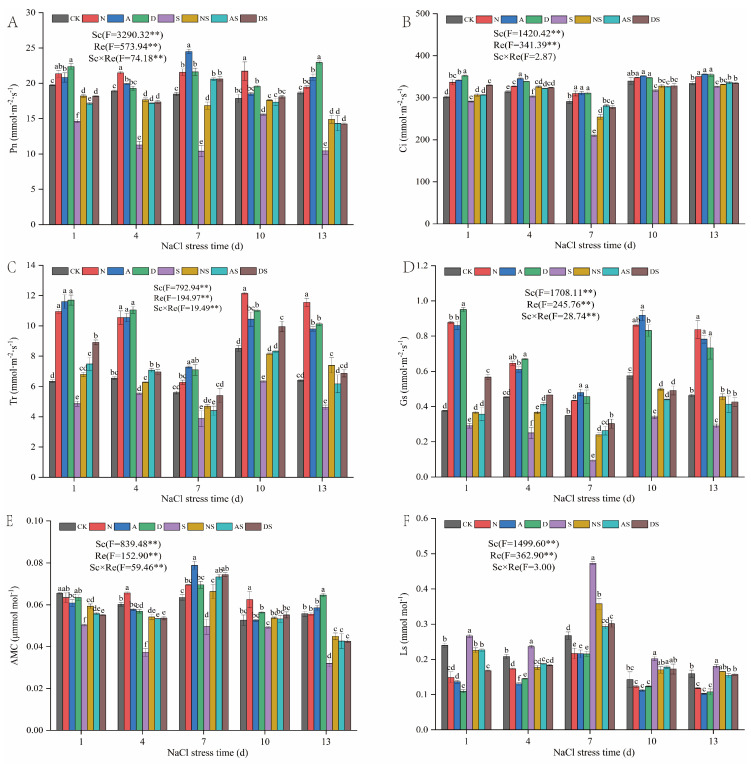
Effect of nitrogen fertilizer and PGRs on gas exchange parameters in rice. (**A**) The content of Pn. (**B**) The content of Ci. (**C**) The content of Tr. (**D**) The content of Gs. (**E**) The content of AMC. (**F**) The content of Ls. CK: 0% NaCl; N: 0% NaCl + 0.05 g N; A: 0% NaCl + 40 mg·L^−1^ 5-ALA; D: 0% NaCl + 30 mg·L^−1^ DTA-6; S: 0.3% NaCl; NS: N + 0.3% NaCl; AS: 40 mg·L^−1^ 5-ALA + 0.3% NaCl; DS: 30 mg·L^−1^ DTA-6 + 0.3% NaCl. Data are shown as mean ± standard error of three replicates. Different lowercase letters in the same column indicate significant differences a(*p*-value = 0.05) between treatments. Sc: salt concentration (0% NaCl and 0.3% NaCl). Re: different regulators (CK, N, 5-ALA, and DTA-6). Sc × Re: salt concentration × different regulators. ** indicate 0.01 significance levels.

Salt concentration (Sc) and regulatory factor (Re) significantly affected Pn, Ci, Tr, Gs, AMC, and Ls (*p* < 0.01) (Figure 2). NaCl stress significantly decreased Pn, Ci, Tr, Gs, and AMC but significantly increased Ls after 1 to 13 days of NaCl stress. Pn, Ci, Tr, Gs, and AMC levels in the S treatment decreased by 12.83–44.02%, 2.53–27.86%, 15.57–30.99%, 22.65–73.32%, and 6.65–42.57%, while Ls increased by 11.21–76.83%. This indicated that NaCl stress inhibited photosynthesis in rice seedlings. Increasing nitrogen fertilizer levels promoted the photosynthesis of the seedlings. In particular, under NaCl stress, Pn, Ci, Tr, Gs, and AMC levels increased by 13.09–62.06%, 1.79–21.23%, 13.63–60.10%, 25.81–157.53%, and 9.36–45.66%, respectively in the NS treatment, whereas Ls decreased by 12.64–37.03%. This indicated that the increased levels of the N treatment reduced the stomatal damage in rice seedling leaves under NaCl stress. In the presence of PGRs, the levels of Pn, Ci, Tr, and Gs increased by 3.61% to 32.60%, 3.51% to 13.38%, 22.74% to 82.75%, and 37.66% to 128.81%, respectively, whereas Ls decreased by 19.17% to 43.02% in the A treatment under NaCl stress (1–13 days). Pn, Ci, Tr, Gs, and AMC increased by 11.39–98.10%, 6.38–33.85%, 13.85–54.10%, 22.61–183.12%, and 8.32–48.05%, respectively, in the A treatment when compared to CK, whereas Ls decreased by 11.65% to 38.12% in the AS treatment when compared to the S treatment. Pn, Ci, Tr, and Gs levels increased by 1.97–23.06%, 2.21–16.86%, 26.68–84.30%, and 30.95–152.84%, respectively, while Ls decreased by 13.39–54.31% in the D treatment when compared to CK. DS treatment also increased Pn, Ci, Tr, Gs, and AMC levels and decreased Ls from day 1 to day 13 after NaCl stress. Pn, Ci, Tr, Gs, and AMC levels increased by 16.13–98.41%, 2.74–32.05%, 25.97–83.33%, 44.03–225.81%, and 9.58–50.27%, respectively, in the DS treatment when compared to the S treatment. The Ls of the DS treatment reduced by 12.64–37.03% when compared to the S treatment. This indicated that spraying with two PGRs promoted seedling photosynthesis and alleviated the photosynthetic damage induced by NaCl stress. Exposure to both PGRs led to an increase in nitrogen fertilizer treatment in promoting rice foliar photosynthesis, especially in ameliorating the photosynthetic damage under NaCl stress. The effect of 5-ALA was slightly better than that of DTA-6.

### 3.3. Effects of 5-ALA, DTA-6, and Nitrogen Application on H_2_O_2_ and O_2_^−^ Distribution in Rice Seedling Leaves

**Figure 3:**
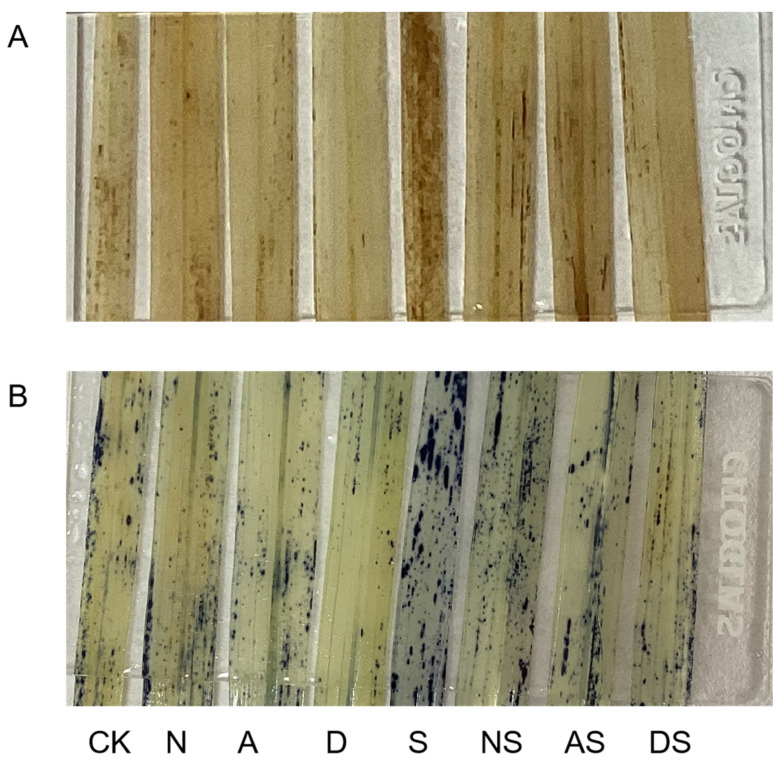
Effects of PGRs and nitrogen fertilizer on H_2_O_2_ and O_2_·^−^ distribution in rice seedling leaves (7 d). (**A**) Distribution of H_2_O_2_. (**B**) Distribution of O_2_^−^. CK: 0% NaCl; N: 0% NaCl + 0.05 g N; A: 0% NaCl + 40 mg·L^−1^ 5-ALA; D: 0% NaCl + 30 mg·L^−1^ DTA-6; S: 0.3% NaCl; NS: N + 0.3% NaCl; AS: 40 mg·L^−1^ 5-ALA + 0.3% NaCl; DS: 30 mg·L^−1^ DTA-6 + 0.3% NaCl.

In this experiment, tissue staining of rice leaves was performed in order to visualize the distribution of H_2_O_2_ and superoxide anion (O_2_·^−^) levels. As shown in Figure 3, H_2_O_2_ and O_2_·^−^ were identified as dark-brown and dark-blue spots in leaves, respectively. As shown in Figure 3A, fewer dark brown spots were found in leaves in the N, A, and D treatments than in leaves in the CK treatment. Salt treatment alone (S) resulted in a significant increase in dark-brown spots of rice leaves when compared to the CK treatment. NS, AS, and DS treatments resulted in significantly fewer dark-brown spots in leaves compared to the S treatment, suggesting that both nitrogen fertilizer and exogenous PGRs effectively reduced the accumulation and distribution of H_2_O_2_ in rice leaves. As shown in Figure 3B, fewer dark-blue spots were observed in leaves in the N, A, and D treatments than in the leaves in the CK treatment. Salt treatment alone (S) resulted in a significant increase in dark-blue spots in rice leaves when compared to the CK treatment. The dark-blue spots in leaves in the NS, AS, and DS treatments were significantly reduced when compared to those in the S treatment. This indicated that both nitrogen fertilizer and exogenous PGRs effectively reduced the accumulation and distribution of O_2_·^−^ in rice leaves. Nitrogen fertilizer and PGRs were equally effective in reducing H_2_O_2_ and O_2_·^−^ accumulation in leaves, and DTA-6 was better than 5-ALA in scavenging ROS.

### 3.4. Effects of 5-ALA, DTA-6, and Nitrogen Treatment on Osmoregulatory Substances in Rice Seedlings

**Figure 4:**
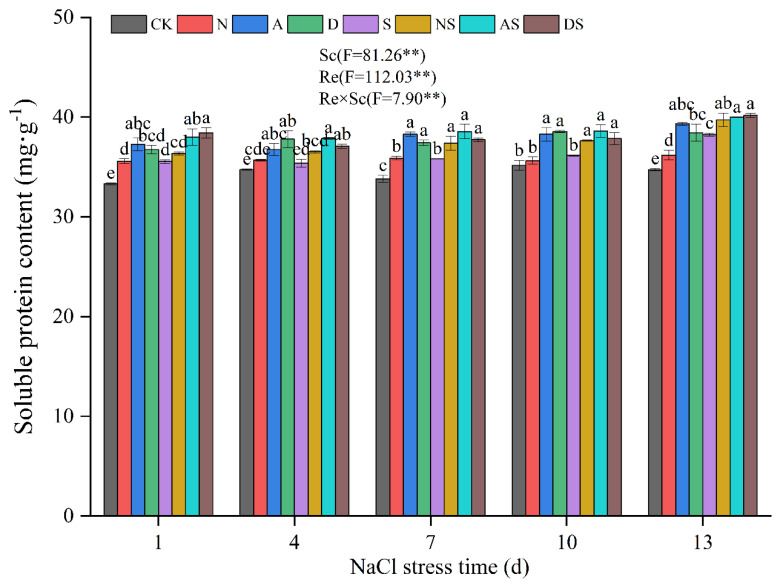
Effects of 5-ALA, DTA-6, and nitrogen fertilizer on soluble protein content in rice. CK: 0% NaCl; N: 0% NaCl + 0.05 g N; A: 0% NaCl + 40 mg·L^−1^ 5-ALA; D: 0% NaCl + 30 mg·L^−1^ DTA-6; S: 0.3% NaCl; NS: N + 0.3% NaCl; AS: 40 mg·L^−1^ 5-ALA + 0.3% NaCl; DS: 30 mg·L^−1^ DTA-6 + 0.3% NaCl. Data are shown as mean ± standard error of three replicates. Different lowercase letters in the same column indicate significant differences (*p*-value = 0.05) between treatments. Sc: salt concentration (0% NaCl and 0.3% NaCl). Re: different regulators (CK, N, 5-ALA, and DTA-6). Sc × Re: salt concentration × different regulators. ** indicate 0.01 significance levels.

As shown in Figure 4, both Sc and Re had a significant effect on soluble protein content (*p* < 0.01). NaCl stress increased the content of soluble proteins. Following 1 to 13 days of NaCl stress, the soluble protein content in the S treatment increased by 1.82–10.11% when compared to levels in the CK treatment. It indicated that NaCl stress induced a self-protective mechanism in rice. The interaction between Sc and Re had a significant effect on soluble protein content (*p* < 0.01). The increased application of nitrogen fertilizer promoted the accumulation of osmoregulatory substances. Under NaCl stress, the soluble protein content in the NS treatment increased by 2.26–4.38% compared to the S treatment. This indicated that the increased application of nitrogen fertilizer alleviated the osmotic stress of NaCl on rice. In the presence of PGRs, exposure to 1 to 13 days of NaCl stress led to an increase in the soluble protein content in the A treatment by 5.75–13.24% when compared to the CK treatment. The soluble protein content in the AS treatment was increased by 4.58–7.61% compared to the S treatment levels. The soluble protein content in the D treatment increased by 8.74–10.66% compared to the CK treatment. Following exposure of 1 to 13 days of NaCl stress, the DS treatment also increased the soluble protein content. The soluble protein content in the DS treatment increased by 4.68–8.09% when compared to these levels in the S treatment. Thus, the spraying of two PGRs promoted the accumulation of soluble proteins. Both PGRs were equally effective in increasing the osmoregulatory efficiency of nitrogen fertilizer under NaCl stress.

### 3.5. Effects of 5-ALA, DTA-6, and Nitrogen Application on Non-Enzymatic Antioxidants and Key Enzyme Activities in the AsA-GSH Cycle of Rice Seedlings

#### 3.5.1. Effects of PGRs and Nitrogen Fertilizer on Non-Enzymatic Antioxidant Levels in AsA-GSH Cycle of Rice Seedlings

**Figure 5:**
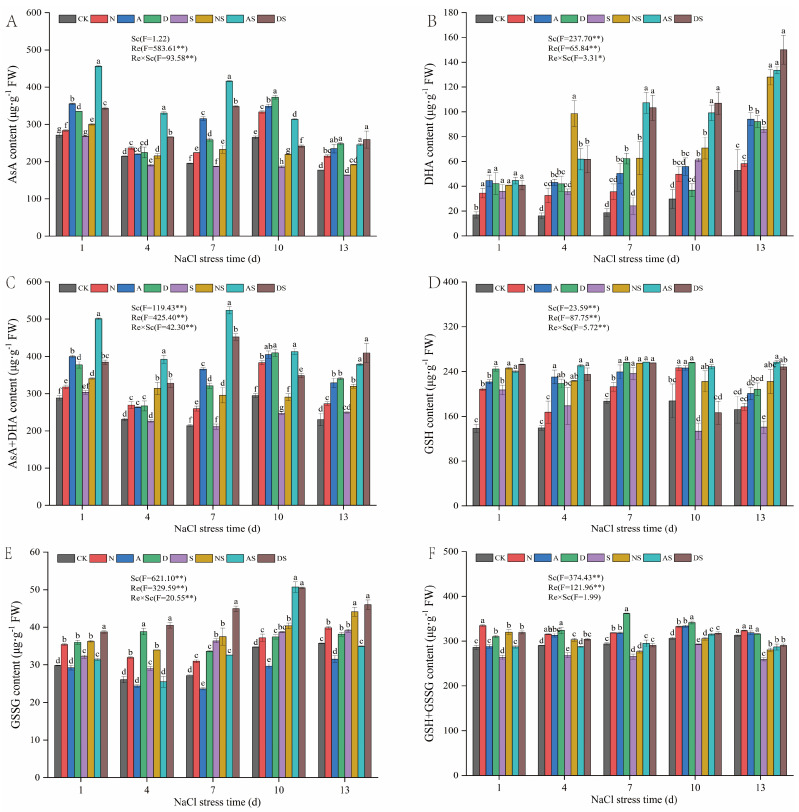
Effects of nitrogen fertilizer 5-ALA and DTA-6 on the AsA and GSH content of rice leaves under NaCl stress. (**A**) The content of AsA. (**B**) The content of DHA. (**C**) The content of AsA + DHA. (**D**) The content of GSH. (**E**) The content of GSSG. (**F**) The content of GSH + GSSG. CK: 0% NaCl; N: 0% NaCl + 0.05 g N; A: 0% NaCl + 40 mg·L^−1^ 5-ALA; D: 0% NaCl + 30 mg·L^−1^ DTA-6; S: 0.3% NaCl; NS: N + 0.3% NaCl; AS: 40 mg·L^−1^ 5-ALA + 0.3% NaCl; DS: 30 mg·L^−1^ DTA-6 + 0.3% NaCl. Data are shown as mean ± standard error of three replicates. Different lowercase letters in the same column indicate significant differences (*p*-value = 0.05) between treatments. Sc: salt concentration (0% NaCl and 0.3% NaCl). Re: different regulators (CK, N, 5-ALA, and DTA-6). Sc × Re: salt concentration × different regulators. ** and * indicate 0.01 and 0.05 significance levels, respectively.

As shown in Figure 5, both the Re and the interaction between Re and Sc significantly affected the levels of AsA, DHA, and their combination (*p* < 0.01). In the S treatment alone, the AsA levels in rice leaves eventually decreased, and the levels of DHA and AsA + DHA combination first decreased and then increased under prolonged NaCl stress. NaCl stress decreased the AsA levels and increased the DHA content in rice (Figure 5A–C). Following 1 to 13 days of NaCl stress, the AsA levels decreased by 1.35–29.82% in the S treatment when compared to the CK treatment, whereas the DHA content increased by 29.35–120.59% in the S treatment when compared to the CK treatment. Increased application of nitrogen fertilizer promoted seedling growth. The levels of AsA, DHA, and AsA + DHA combination in the N treatment were increased by 4.53–25.79%, 10.18–104.16%, and 10.38–29.95%, respectively, when compared to the CK treatment. Under NaCl stress, AsA, DHA, and AsA + DHA levels increased by 12.12–24.64%, 13.85–176.48%, and 12.33–40.01%, respectively, in rice treated with NS compared to S. This indicates that the increased application of nitrogen fertilizer increases the AsA content of rice seedling leaves. Exposure to PGRs from day 1 to day 13 d after NaCl stress led to an increase in the levels of AsA, DHA, and ASA + DHA in the A treatment by 2.63–61.57%, 78.14–168.53%, and 14.11–70.95%, respectively, when compared to that of CK, while the levels of AsA, DHA, and ASA + DHA in the AS treatment increased by 49.75–122.61%, 24.51–342.08%, and 51.86–147.83%, respectively. The AsA, DHA, and AsA + DHA levels of rice treated with D increased by 4.68–40.76%, 23.67–232.35%, and 15.59–50.02%, respectively, when compared to that of CK. The AsA, DHA, and AsA + DHA levels in the DS treatment increased significantly by 28.32–86.42%, 14.01–325.51%, and 26.63–113.90%, respectively, when compared to that of S treatment after 1 to 13 days of NaCl stress. The levels of ASA, DHA, and AsA + DHA increased in the AS and DS treatments when compared to the NS treatment, following 7 to 13 days of NaCl stress, with significant increases of 28.06–78.60% and 9.93–49.56% in ASA, 4.31–71.11% and 17.22–64.70% in DHA, and a significant increase in AsA + DHA by 18.52–77.01% and 19.95–52.77%. Thus, the spraying of two PGRs increased AsA levels and thus reduced the damage caused by NaCl stress. The two PGRs increased the AsA levels in rice under NaCl stress in proportion to the increase in the quantity of nitrogen fertilizer used.

As can also be seen in Figure 5, both Sc and Re significantly affected the levels of GSH, GSSG, and GSH + GSSG (*p* < 0.01). NaCl stress increased and then decreased GSH content, increased GSSG content, and decreased GSH + GSSG levels in rice (Figure 5D–F). Following 1–7 days of NaCl stress, the GSH levels in the S treatment increased by 26.47–49.63% compared to the CK treatment. Following 10 and 14 days of exposure to NaCl stress, the GSH content of the S treatment decreased by 28.81% and 18.18%, respectively, compared to the CK treatment. Under NaCl stress, the GSSG content of the S treatment significantly increased by 7.91–34.12% when compared to the CK treatment, whereas the GSH + GSSG levels in the S treatment decreased significantly by 4.45–17.15% when compared to the CK treatment. The added application of nitrogen fertilizer increased the levels of GSH. GSH, GSSG, and GSH + GSSG levels in the N treatment increased by 3.02–50.52%, 7.15–22.61%, and 3.40–17.03%, respectively, when compared to the CK treatment. Under NaCl stress, GSH, GSSG, and GSH + GSSG levels increased by 7.7–66.72%, 3.16–16.94%, and 4.14–21.45%, respectively, in the NS treatment when compared to the S treatment. This indicates that increased exposure to nitrogen fertilizer increased the GSH content of rice. Exposure to PGRs between days 1 and 13 d after NaCl stress led to an increase in the GSH and GSH + GSSG levels in the A treatment by 16.82–65.44% and 0.76–8.79%, respectively, while the GSSG levels decreased by 1.95–14.59% when compared to the CK treatment. The GSH and GSH + GSSG levels in the AS treatment increased by 8.58–86.42% and 7.06–11.17%, while the GSSG content decreased. The GSH, GSSG, and GSH + GSSG levels in the D treatment increased by 21.22–76.68%, 6.75–49.10%, and 8.54–23.15%, respectively, when compared to the CK. The GSH, GSSG, and GSH + GSSG levels in the DS treatment increased by 8.02–76.52%, 17.69–39.63%, and 8.46–21.18%, respectively, when compared to the S treatment, between days 1 and 13 after NaCl stress. This indicates that the spraying of the two PGRs increased GSH content, and the magnitude of the increase under NaCl stress was comparable to the effect of the nitrogen fertilizer.

#### 3.5.2. Effects of 5-ALA, DTA-6, and Nitrogen Fertilizer on the Activities of Key Enzymes in the AsA-GSH Cycle of Rice

**Figure 6:**
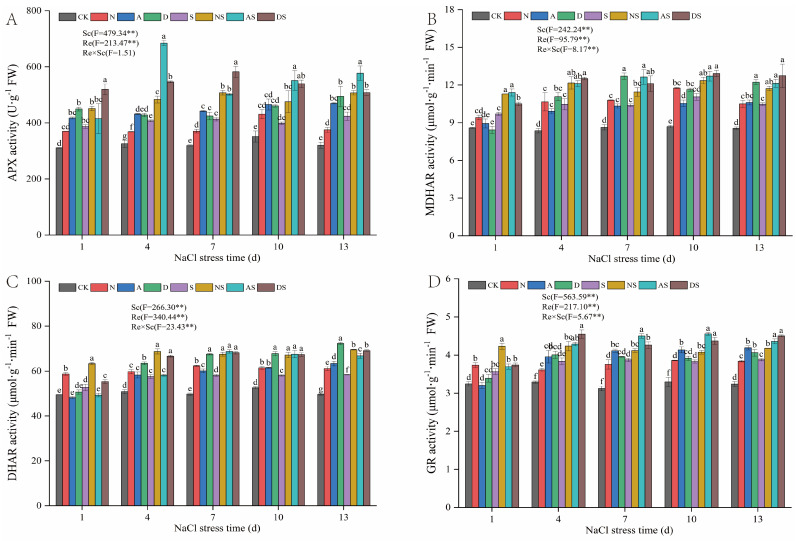
Effects of nitrogen fertilization and PGRs on the activities of key enzymes in the AsA-GSH cycle of rice leaves under NaCl stress. (**A**) The activity of APX. (**B**) The activity of MDHAR. (**C**) The activity of DHAR. (**D**) The activity of GR. CK: 0% NaCl; N: 0% NaCl + 0.05 g N; A: 0% NaCl + 40 mg·L^−1^ 5-ALA; D: 0% NaCl + 30 mg·L^−1^ DTA-6; S: 0.3% NaCl; NS: N + 0.3% NaCl; AS: 40 mg·L^−1^ 5-ALA + 0.3% NaCl; DS: 30 mg·L^−1^ DTA-6 + 0.3% NaCl. Data are shown as mean ± standard error of three replicates. Different lowercase letters in the same column indicate significant differences (*p*-value = 0.05) between treatments. Sc: salt concentration (0% NaCl and 0.3% NaCl). Re: different regulators (CK, N, 5-ALA, and DTA-6). Sc × Re: salt concentration × different regulators. ** indicate 0.01 significance levels.

As shown in Figure 6, Sc and Re significantly affected APX, MDHAR, DHAR, and GR activities (*p* < 0.01). NaCl stress significantly increased APX, MDHAR, DHAR, and GR activities in rice. APX, MDHAR, DHAR, and GR activities increased by 13.35–29.38%, 12.81–27.21%, 6.57–17.54%, and 10.04–24.04%, respectively, from days 1 to 13 after NaCl stress, in the S treatment when compared to the CK treatment. Increased nitrogen fertilizer levels significantly increased the activities of key enzymes in the AsA-GSH cycle. APX, MDHAR, DHAR, and GR activities increased by 13.28–22.55%, 9.57–35.33%, 16.83–25.33%, and 9.73–20.25% in the N treatment when compared to the CK treatment, respectively—under NaCl stress, APX, MDHAR, DHAR, and GR activities significantly increased by 16.70–22.85%, 10.34–16.42%, 15.70–20.19% and 6.33–18.51%, respectively, in the NS treatment when compared to the S treatment. This indicates that increased nitrogen fertilizer levels can increase key enzyme activities in the AsA-GSH cycle in rice. Exposure to PGRs resulted in a significant elevation in APX, MDHAR, DHAR, and GR activities by 32.40–46.77%, 19.52–24.10%, 16.90–27.58%, and 25.53–31.69%, respectively, in the A treatment when compared to the CK treatment from days 7 to 13 after NaCl stress. APX, MDHAR, DHAR, and GR activities significantly increased by 21.53–38.25%, 14.93–21.81%, 14.40–18.38%, and 12.340–18.76%, respectively, in the AS treatment when compared to the S treatment. APX, MDHAR, DHAR, and GR activities significantly increased by 31.07–54.41%, 32.46–47.15%, 24.96–45.37%, and 18.92–26.65%, respectively, from day 4 to day 13 after NaCl stress, in the D treatment when compared to the CK treatment. The APX, MDHAR, DHAR, and GR activities progressively increased by 19.79–40.99%, 16.68–21.60%, 15.99–18.33%, and 9.90–16.07%, respectively, between days 7 and 13 after NaCl stress, in the DS treatment when compared to the S treatment areas. This indicated that the spraying of both PGRs increased key enzyme activities of the AsA-GSH cycle, and the magnitude of both PGRs in increasing these in salt-stressed rice was quantitatively comparable to the effect of increasing nitrogen fertilizer levels.

### 3.6. Effects of 5-ALA, DTA-6, and Nitrogen Application on Carbon Metabolism in Rice

#### 3.6.1. Effect of PGRs and Nitrogen Fertilizer on Major Carbohydrate Content of Rice Seedlings

**Figure 7:**
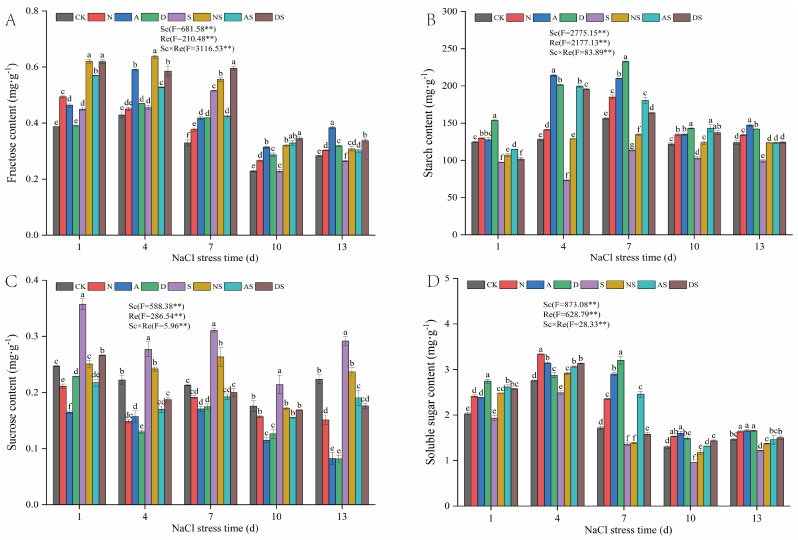
Effects of nitrogen fertilization and PGRs on the carbohydrate content of rice leaves under NaCl stress. (**A**) The content of Fructose. (**B**) The content of Starch. (**C**) The content of Sucrose. (**D**) The content of Soluble. CK: 0% NaCl; N: 0% NaCl + 0.05 g N; A: 0% NaCl + 40 mg·L^−1^ 5-ALA; D: 0% NaCl + 30 mg·L^−1^ DTA-6; S: 0.3% NaCl; NS: N + 0.3% NaCl; AS: 40 mg·L^−1^ 5-ALA + 0.3% NaCl; DS: 30 mg·L^−1^ DTA-6 + 0.3% NaCl. Data are shown as mean ± standard error of three replicates. Different lowercase letters in the same column indicate significant differences (*p*-value = 0.05 level) between treatments. Sc: salt concentration (0% NaCl and 0.3% NaCl). Re: different regulators (CK, N, 5-ALA, and DTA-6). Sc × Re: salt concentration × different regulators. ** indicate 0.01 significance levels.

As can be seen in Figure 7, Sc and Re significantly affected the fructose, starch, sucrose, and soluble sugar levels (*p* < 0.01). NaCl stress increased and then decreased fructose content, decreased starch and soluble sugar levels, and increased sucrose content in rice. The fructose content in the S treatment increased by 6.27–56.53% when compared to the CK treatment after 1 to 7 days of NaCl stress. Between days 10 and 13 of NaCl stress, the fructose content in the S treatment reduced by 0.09% and 6.65%, respectively, when compared to the CK treatment. Under NaCl stress, starch and soluble sugar levels were reduced by 15.45–42.75% and 4.65–26.17%, respectively, in the S treatment when compared to the CK treatment. The S treatment significantly increased sucrose content by 21.94% to 45.80% compared to the CK treatment. The Sc × Re interaction significantly affected fructose, starch, sucrose, and soluble sugar levels (*p* < 0.01). Increased nitrogen fertilizer application increased the levels of fructose, starch, and soluble sugar and reduced sucrose levels. Especially under NaCl stress, the levels of fructose, starch, and soluble sugar increased by 7.84–40.24%, 9.92–76.01%, and 2.01–28.51%, respectively, in the NS treatment when compared to the S treatment and the sucrose content significantly reduced by 12.66–29.79% in the NS treatment when compared to the S treatment. Both PGRs increased the concentrations of fructose, starch, and soluble sugar and decreased sucrose content. The levels of fructose, starch, and soluble sugar increased by 19.87–37.70%, 2.36–67.62%, and 13.02–68.71%, respectively, from days 1 to 13 after NaCl stress, whereas the sucrose content decreased by 19.80–63.27% in the A treatment when compared to the CK treatment. The starch and soluble sugar levels increased by 18.09–172.45% and 20.02–80.85%, respectively, in the AS treatment when compared to the S treatment, and the sucrose content significantly reduced by 27.65% to 39.03% in the AS treatment when compared to the S treatment. The levels of fructose, starch, and soluble sugar increased by 0.83–27.22%, 14.61–57.56%, and 4.18–86.40%, respectively, from days 1 to 13 d after NaCl stress, while sucrose content decreased by 7.32–63.54% in the D treatment when compared to the CK treatment. Fructose, starch, and soluble sugar levels increased by 15.64–50.83%, 4.49–167.42%, and 16.15–49.45%, respectively, in the DS treatment when compared to the S treatment. The sucrose content was significantly reduced by 21.17% to 39.55% in the DS treatment compared to the S treatment. Both PGRs effectively regulated fructose, starch, sucrose, and soluble sugar levels to increase nitrogen fertilizer use.

#### 3.6.2. Effects of 5-ALA, DTA-6, and Nitrogen Fertilizer on Enzyme Activities Related to Carbon Metabolism in Rice

**Figure 8:**
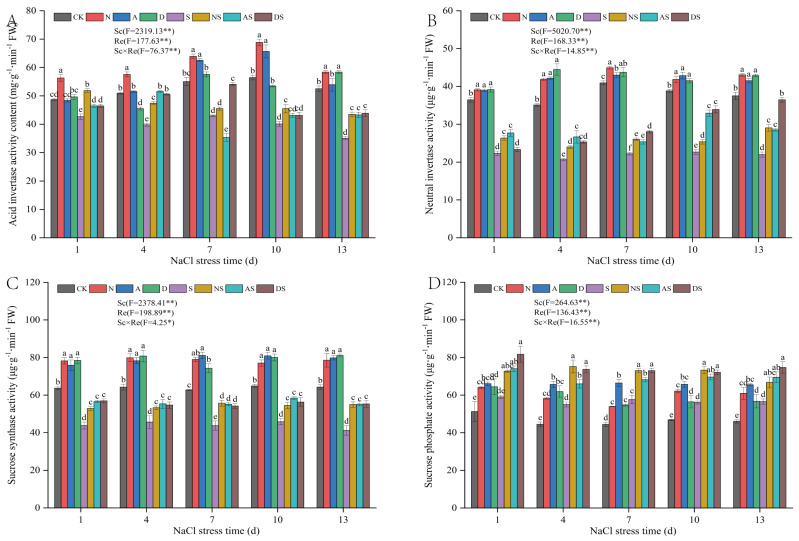
Effects of nitrogen fertilizer and PGRs on the activities of enzymes related to sucrose metabolism in rice leaves under NaCl stress. (**A**) The activity of Acid invertase. (**B**) The activity of neutral invertase. (**C**) The activity of Sucrose synthase. (**D**) The activity of Sucrose phosphate. CK: 0% NaCl; N: 0% NaCl + 0.05 g N; A: 0% NaCl + 40 mg·L^−1^ 5-ALA; D: 0% NaCl + 30 mg·L^−1^ DTA-6; S: 0.3% NaCl; NS: N + 0.3% NaCl; AS: 40 mg·L^−1^ 5-ALA + 0.3% NaCl; DS: 30 mg·L^−1^ DTA-6 + 0.3% NaCl. Data are shown as mean ± standard error of three replicates. Different lowercase letters in the same column indicate significant differences (*p*-value = 0.05) between treatments. Sc: salt concentration (0% NaCl and 0.3% NaCl). Re: different regulators (CK, N, 5-ALA, and DTA-6). Sc × Re: salt concentration × different regulators. ** indicate 0.01significance levels.

As shown in Figure 8, Sc and Re significantly affected the activities of AI, NI, SS, and SPS (*p* < 0.01). NaCl stress decreased the activities of AI, NI, and SS in rice and increased the activity of SPS. Between days 1 and 13 of NaCl stress, the activities of AI, NI, and SS were significantly reduced by 12.39–33.25%, 38.78–45.81%, and 28.97–35.92% in the S treatment when compared to the CK treatment, while the activities of SPS were increased by 15.45–29.76% in the S treatment when compared to the CK treatment. The Sc × Re interactions significantly affected AI, NI, SS, and SPS activities (*p* < 0.05). Increased application of nitrogen fertilizer led to a significant increase in AI, NI, SS, and SPS activities between days 1 and 13 under NaCl stress by 11.19–21.92%, 7.26–19.18%, 18.84–25.98%, and 21.33–32.77% when compared to the CK treatment, respectively. AI, NI, SS, and SPS activities significantly increased by 5.91–24.00%, 11.89–32.16%, 16.70–33.42%, and 18.09–30.82%, respectively, in the NS treatment when compared to the S treatment. Both plant growth regulators significantly increased the AI, NI, SS, and SPS activities from days 1 to 13 of NaCl stress by 5.02–20.13%, 19.18–29.44%, and 28.90–49.66%, respectively, in the A treatment when compared to the CK treatment. NI, SS, and SPS activities were significantly increased in the AS treatment when compared to the S treatment by 14.26–45.32%, 21.21–33.53%, and 18.36–25.41%, respectively. NI, SS, and SPS activities significantly increased by 6.83–26.65%, 18.50–26.15%, and 20.58–38.99%, respectively, in the D treatment compared to the CK treatment, from day 1 to 13 under NaCl stress. DS treatment led to a significant increase in NI, SS, and SPS activities by 4.52–65.85%, 19.77–34.13%, and 26.71–38.50, respectively. Exposure to both PGRs regulated the AI, NI, SS, and SPS activities in proportion to the extent of the increase in nitrogen fertilizer levels.

**Figure 9:**
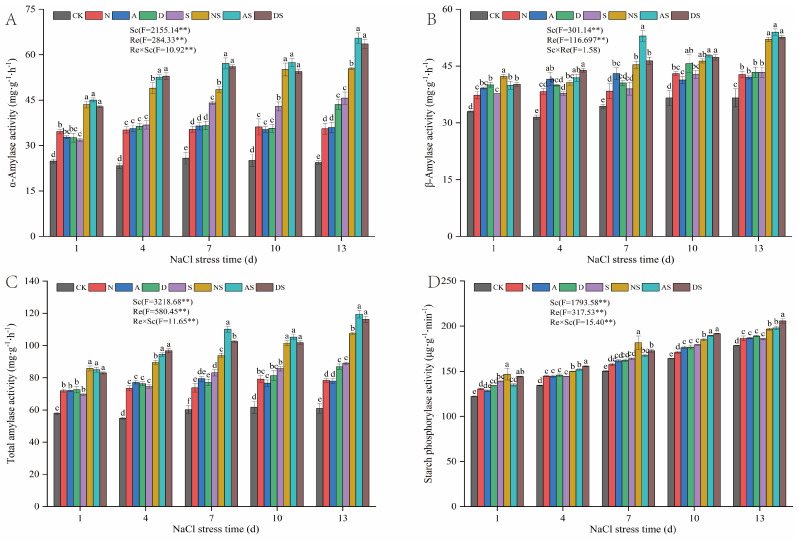
Effects of nitrogen fertilizer and PGRs on the activities of enzymes related to starch metabolism in rice leaves under NaCl stress. (**A**) The activity of α-amylase. (**B**) The activity of β-amylase. (**C**) The activity of Total amylase. (**D**) The activity of Starch phosphorylase. CK: 0% NaCl; N: 0% NaCl + 0.05 g N; A: 0% NaCl + 40 mg·L^−1^ 5-ALA; D: 0% NaCl + 30 mg·L^−1^ DTA-6; S: 0.3% NaCl; NS: N + 0.3% NaCl; AS: 40 mg·L^−1^ 5-ALA + 0.3% NaCl; DS: 30 mg·L^−1^ DTA-6 + 0.3% NaCl. Data are shown as mean ± standard error of three replicates. Different lowercase letters in the same column indicate significant differences (*p*-value = 0.05) between treatments. Sc: salt concentration (0% NaCl and 0.3% NaCl). Re: different regulators (CK, N, 5-ALA, and DTA-6). Sc × Re: salt concentration × different regulators. ** indicate 0.01 significance levels.

As can be seen in Figure 9, Sc and Re significantly affected the activity of α-amylase, β-amylase, total amylase, and starch phosphorylase (SP) (*p* < 0.01). NaCl stress increased the activities of α-amylase, β-amylase, total amylase, and SP in rice. Between days 1 and 13 of NaCl stress, the activities of α-amylase, β-amylase, total amylase, and SP were significantly increased by 27.99–87.14%, 13.51–19.86%, 20.18–45.92%, and 4.13–13.55% in the S treatment when compared to those in the CK treatment. The Sc × Re interactions partially affected the activities of α-amylase, β-amylase, total amylase, and SP, although these effects were not significant. The added application of nitrogen fertilizer increased the activities of α-amylase, β-amylase, total amylase, and SP by 36.66–50.61%, 13.20–21.57%, 22.45–33.93%, and 4.26–7.71%, respectively, in the N treatment when compared to the CK treatment, between days 1 and 13 of NaCl stress. The α-amylase, β-amylase, total amylase, and SP activities increased by 10.01–36.64%, 7.54–20.09%, 12.96–23.38%, and 3.16–10.73%, respectively, in the NS treatment when compared to the S treatment. Both PGRs significantly increased the α-amylase, β-amylase, total amylase, and SP activities by 31.61–52.53%, 14.91–31.85%, 24.29–27.88%, and 4.73–7.61%, respectively, in the A treatment when compared to the CK treatment. α-Amylase, β-amylase, and total amylase activities significantly increased by 29.58%~43.35%, 5.91%~35.75%, and 22.17%~34.19%, respectively, in the AS treatment when compared to the S treatment. These enzyme activities significantly increased by 30.92–78.48%, 17.95–26.83%, 25.50–42.49%, and 5.93–9.81%, respectively, in the D treatment when compared to the CK treatment. The enzyme activities significantly increased by 27.00–43.71%, 6.59–21.49%, and 3.99–10.78%, respectively, in the DS treatment when compared to the S treatment by 27.00–43.71%, 6.59–21.49%, 18.83–30.55% and 3.99–10.78%, respectively. The two PGRs were comparable in the regulation of α-amylase, β-amylase, total amylase, and SP activities and exposure to additional nitrogen fertilizer. The regulatory effect of 5-ALA was slightly better than that of DTA-6.

## 4. Discussion

### 4.1. Effects of NaCl Stress on Rice Seedling Morphology and the Regulatory Effects of PGRs and Nitrogen Fertilizer

NaCl stress inhibits plant growth, reduces plant leaf area, decreases plant biomass, and may even lead to plant death. Inhibition of crop growth by NaCl stress has been reported in a variety of crops, such as rice [49,50], wheat [51], and sweet sorghum [52]. Nitrogen (N), an important component of plant protein, has an essential role in rice cultivation [53,54]. Previous studies have shown that the addition of urea to the soil can promote rice growth and thus increase rice yield [55]. The results of this study indicate that the morphological indices (plant height, leaf area, aboveground fresh weight, and aboveground dry weight) of rice seedlings show a decreasing trend under prolonged NaCl stress, indicating that NaCl stress inhibits seedling growth. Under NaCl stress, both nitrogen fertilizer treatment and foliar spraying of both PGRs improves the salt tolerance of seedlings, increasing seedling height, leaf area, and aboveground fresh weight, and dry weight (Figure 1 and Table 2). Thus, seedling growth was enhanced and NaCl stress was partially alleviated, which is consistent with a previous study reporting that the exposure to nitrogen fertilizer, 5-ALA, and DTA-6 promoted plant growth under adverse conditions [56]. Overall, 5-ALA appears to be slightly superior to DTA-6 in improving rice morphology, and the effects of leaf spraying of both GPRs on rice seedling morphology were comparable to those of an additional 0.05 g of pure nitrogen per pot.

### 4.2. Effects of NaCl Stress on Photosynthesis and Osmoregulatory Substances in Rice and Regulatory Effects of PGRs and Nitrogen Fertilizer

Maintaining plant photosynthesis is an important mechanism to improve plant salt tolerance [57]. Salt stress suppresses net photosynthetic rate (Pn) via stomatal or non-stomatal limiting factors [58]. This study showed that Ci, Tr, and Gs rice leaves decreased, whereas Ls increased under NaCl stress, indicating that the decrease in Pn was mainly caused by stomatal limiting factors (Figure 2). Some studies have shown that NaCl stress damages chloroplast structure and reduces chlorophyll content, leading to reduced photosynthesis and plant physiology [59]. Therefore, the decrease in the content of various photosynthetic pigments under salt stress may also be one of the underlying factors for a decrease in the photosynthetic rate. The results of this study showed that exposure to 5-ALA, DTA-6, and nitrogen fertilizer resulted in high levels of Gs, Tr, and Ci and reduced Ls in rice leaves. The increase in Gs facilitated the entry of CO_2_ into rice leaves for gas exchange and carbon assimilation. This suggests that 5-ALA, DTA-6, and nitrogen fertilizer contribute to the maintenance of photosynthetic light reactions in leaves, which is consistent with previous studies suggesting that these treatments improve photosynthetic energy conversion efficiency [60]. In this study, we showed that the effects of two leaf spray regulators on photosynthetic parameters under NaCl stress were comparable to those of increasing quantitative nitrogen fertilizer, with 5-ALA being more effective than DTA-6. This may be attributed to 5-ALA being a common precursor for the biosynthesis of all porphyrin compounds. 5-ALA directly participates in chlorophyll synthesis and maintains normal electron transfer in the photosynthetic system [61]. Interestingly, in this study, the overall trends of 5-ALA, DTA-6, and nitrogen fertilizer on the regulation of photosynthetic parameters Pn and AMC over time initially increased and then decreased, which may be due to the fact that the efficacy weakened over time. It is also tempting to speculate that more energy is required to sustain rice growth under prolonged NaCl stress. Furthermore, the influence of the non-stomatal limiting factors cannot be disregarded.

Osmotic stress is one of the major stressors induced by NaCl. Increasing osmoregulatory substances in plants can balance the water potential of vesicles and cytoplasm and thus improve osmotic stress. Soluble protein content reflects the overall metabolic level of plants, and abiotic stress has a high impact on plant protein synthesis [62]. This study showed that NaCl stress increased soluble protein content. We hypothesized that rice seedlings reduced intracellular water potential and prevented excessive cellular water loss by increasing the accumulation of soluble protein content (Figure 2). Under NaCl stress, treatment with nitrogen fertilizer, 5-ALA, and DTA-6 increased the soluble protein content of rice seedlings and the cellular salt tolerance. Both nitrogen fertilizer and PGRs were found to regulate the water potential of rice leaves, maintaining a lower intracellular osmotic potential and stabilizing protein and membrane structures by increasing the content of soluble proteins, thus improving the resistance of rice seedlings to NaCl stress.

### 4.3. Effects of NaCl Stress on Non-Enzymatic Antioxidants and Key Enzyme Activities in Rice and the Regulatory Effects of PGRs and Nitrogen Fertilizer

The antioxidant defense mechanism in plants consists of antioxidant enzymes and non-enzymatic antioxidants. AsA and GSH are key non-enzymatic antioxidants, and the AsA-GSH cycle effectively scavenges metabolically produced H_2_O_2_, which plays a key role in clearing ROS [63]. As an important scavenger of ROS in plants, anti-chemoglobinic acid reduces plant cell damage under salt stress. In this study, NaCl stress decreased the contents of AsA, AsA + DHA, and GSH + GSSG (Figure 5), while the accumulation of H_2_O_2_ and O_2_·^−^ increased (Figure 6). This indicated that NaCl stress weakened the ability of rice seedlings to scavenge ROS. The levels of DHA and GSSG increased under NaCl stress. We hypothesized that rice seedlings self-preserved under adverse conditions to resist damage caused by NaCl stress. Rice also increased DHA reduction to partly eliminate the H_2_O_2_ produced by stress. In this study, nitrogen fertilizer and DTA-6 increased the levels of anthemic acid and glutathione. In contrast, 5-ALA increased the AsA content, which led to an increase in the GSH and GSH + GSSSG levels and a decrease in the GSSG content in glutathione. This may be attributed to the 5-ALA-induced enhancement of GR activity, which in turn facilitated the conversion of GSSG to GSH [64]. APX is an important antioxidant enzyme in the AsA-GSH cycle that catalyzes the oxidative scavenging of O_2_·^−^ and H_2_O_2_ by AsA. MDHAR, DHAR, and GR provide substrates for APX via the regenerative reaction of ASA with GSH [65]. In this study, APX, MDHAR, DHAR, and GR activities were increased under salt stress. Exposure to nitrogen fertilizer and both PGRs further increased APX, MDHAR, DHAR, and GR activities. This suggests that both nitrogen fertilizer and PGRs can promote the regeneration of ASA and GSH in this cycle and accelerate the removal of ROS by AsA, thus improving the salt tolerance of rice. Furthermore, foliar sprays of 5-ALA and DTA-6 increased APX and GR activities more than nitrogen fertilizer at the late stage of NaCl treatment, further improving rice antioxidant capacity by regulating APX and GR activities. In summary, treatment with both nitrogen fertilizer and PGRs can effectively regulate the systematic operation of non-enzymatic antioxidants in the AsA-GSH cycle and comprehensively improve cellular antioxidant capacity.

### 4.4. Effects of NaCl Stress on Carbon Metabolism in Rice and Regulatory Effects of PGRs and Nitrogen Fertilizer

Carbohydrates are important organic metabolites in plants and are the main energy substrates that sustain plant life [66]. Higher carbohydrate content leads to higher plant growth. Fructose, starch, sucrose, and soluble sugars are important components of carbohydrates [67]. AI, NI, SS, and SPS are key enzymes in the sucrose metabolism. SS and SPS promote the conversion of light energy to sugar in plants [68]. In this study, NaCl stress increased the sucrose content, which may be attributed to the increased activity of SPS in sucrose synthesis and the decreased activity of AI and NI, which catalyze sucrose catabolism (Figure 7). NaCl stress initially increased and subsequently decreased fructose content, resulting in lower levels of starch and soluble sugar. The reduction in starch and soluble sugar content implies a decrease in plant carbohydrate reserves, which is detrimental to the basal metabolism of rice seedlings under NaCl stress. This may be related to the inhibition of SS activity under salt stress in this study (Figure 8). In this study, treatment with both nitrogen fertilizer and PGRs led to the decomposition of sucrose to fructose by increasing the activities of AI, NI, and SS, which resulted in a decrease in sucrose and an increase in fructose and soluble sugar levels in rice leaves, thus enhancing the osmotic adaptation of rice (Figure 7 and Figure 8). However, Zhao et al. [69] showed that exogenous PGRs increased the sucrose content by increasing the activities of SS and SPS in maize leaves, which was contrary to what was seen in this study. This may be explained by the coordination of plant carbohydrate metabolism via source-store-flow mechanisms. Sugar accumulation is also strongly linked to substrate transportation. The decrease in sucrose in rice leaves is related to the conversion of sucrose to fructose and the partial translocation of sucrose to other parts of the plant. Under NaCl stress, exposure to both nitrogen fertilizer and exogenous PGRs increased the activities of carbon metabolism-related enzymes, which in turn reduced plant damage due to NaCl stress. The quantitative effects of two PGRs on the regulation of carbon metabolism under salt stress were similar to those of increased nitrogen fertilizer application.

## 5. Conclusions

Salt stress inhibited the growth and metabolism of rice seedlings. Both the nitrogen fertilizer and foliar sprays of 5-ALA or DTA-6 improved the morphology of rice seedlings under NaCl stress, promoted photosynthesis, reduced the accumulation of H_2_O_2_ and O_2_·^−^, increased the carbohydrate content, and improved the activities of key enzymes. As a result, plant injury from NaCl stress was reduced, and the salt tolerance of rice was improved. In this study, we also found that the application of 5-ALA or DTA-6 was more effective in alleviating osmotic stress in seedlings than exposure to additional levels of nitrogen fertilizers. 5-ALA was more effective in alleviating the morphology and antioxidant enzyme activities of seedlings under NaCl stress, whereas DTA-6 was more effective in alleviating membrane damage. This indicates that 5-ALA and DTA-6 are comparable to nitrogen fertilizers in improving the salt tolerance of rice. Thus, we hypothesize that the exogenous application of these two PGRs based on low nitrogen levels can reduce the amount of nitrogen fertilizer applied to rice. Accordingly, the findings serve as a standard of reference to reduce the application of nitrogen fertilizers and increase the efficiency of rice production in the field. These findings provide an important reference for other economic crops.

## Figures and Tables

**Table 1:** Exposure to different treatments, NaCl, and nitrogen dosages.

Treatment	DTA-6 (mg·L^−1^)	5-ALA (mg·L^−1^)	NaCl (%) (*w*/*w*)	N (g/pot)
CK	0	0	0	0
N	0	0	0	0.05
A	0	40	0	0
D	30	0	0	0
S	0	0	0.3	0
NS	0	0	0.3	0.05
AS	0	40	0.3	0
DS	30	0	0.3	0

**Table 2:** Effects of PGRs and nitrogen fertilization on rice seedlings under NaCl stress. CK: 0% NaCl; N: 0% NaCl + 0.05 g N; A: 0% NaCl + 40 mg·L^−1^ 5-ALA; D: 0% NaCl + 30 mg·L^−1^ DTA-6; S: 0.3% NaCl; NS: N + 0.3% NaCl; AS: 40 mg·L^−1^ 5-ALA + 0.3%NaCl; DS: 30 mg·L^−1^ DTA-6 + 0.3% NaCl. Data are shown as mean ± standard error of three replicates. Different lowercase letters in the same column indicate significant differences (*p*-value = 0.05) among treatments. Sc: salt concentration (0% NaCl and 0.3% NaCl). Re: different regulators (CK, N, 5-ALA, and DTA-6). Sc × Re: salt concentration × different regulators. ** and * indicate 0.001 and 0.05 significance levels, respectively.

Morphological Indicators	Treatment	Day after NaCl Treatment (d)				
		1	4	7	10	13
Shoot length (cm)	CK	29.07 ± 0.18 ^bc^	28.03 ± 0.03 ^d^	32.07 ± 0.07 ^d^	34.07 ± 0.15 ^e^	37.63 ± 0.07 ^d^
	N	30.07 ± 0.15 ^a^	29.53 ± 0.09 ^c^	36.43 ± 0.19 ^c^	37.67 ± 0.18 ^bc^	39.07 ± 0.55 ^c^
	A	28.90 ± 0.06 ^bc^	34.40 ± 0.21 ^ab^	38.47 ± 0.09 ^a^	38.43 ± 0.28 ^b^	40.00 ± 0.21 ^b^
	D	30.13 ± 0.37 ^a^	34.10 ± 0.32 ^b^	38.60 ± 0.10 ^a^	39.90 ± 0.20 ^a^	42.50 ± 0.06 ^a^
	S	29.03 ± 0.23 ^bc^	27.30 ± 0.17 ^e^	29.53 ± 0.39 ^e^	33.30 ± 0.12 ^e^	36.03 ± 0.03 ^e^
	NS	29.60 ± 0.31 ^ab^	29.17 ± 0.12 ^c^	35.67 ± 0.58 ^c^	36.67 ± 0.24 ^d^	37.20 ± 0.15 ^d^
	AS	28.67 ± 0.33 ^c^	34.83 ± 0.17 ^a^	37.43 ± 0.15 ^b^	37.50 ± 0.50 ^c^	42.70 ± 0.15 ^a^
	DS	29.97 ± 0.19 ^a^	34.53 ± 0.03 ^ab^	37.63 ± 0.07 ^b^	39.63 ± 0.27 ^a^	39.93 ± 0.07 ^b^
	F-value	Sc (F = 21.50 **) Re (F = 281.69 **) Sc × Re (F = 4.4 *)				
Leaf area (cm^2^)	CK	935.23 ± 15.30 ^bc^	1075.83 ± 9.22 ^bc^	1154.77 ± 6.20 ^bc^	1251.67 ± 21.95 ^b^	1368.53 ± 18.73 ^d^
	N	1080.40 ± 25.03 ^a^	1106.97 ± 14.11 ^ab^	1266.23 ± 25.20 ^a^	1430.83 ± 48.39 ^a^	1518.20 ± 7.37 ^c^
	A	830.17 ± 12.86 ^d^	1116.10 ± 26.68 ^ab^	1204.6 ± 31.22 ^abc^	1393.97 ± 54.58 ^a^	1676.30 ± 28.02 ^a^
	D	960.67 ± 8.04 ^b^	1174.23 ± 21.18 ^a^	1254.03 ± 57.62 ^ab^	1454.23 ± 29.72 ^a^	1599.03 ± 11.71 ^b^
	S	827.03 ± 8.783 ^d^	857.93 ± 11.07 ^d^	946.53 ± 32.59 ^d^	1044.90 ± 7.97 ^c^	1147.50 ± 25.67 ^f^
	NS	928.70 ± 61.55 ^bc^	1045.03 ± 14.50 ^bc^	1108.20 ± 50.34 ^c^	1163.00 ± 11.09 ^bc^	1250.47 ± 35.81 ^e^
	AS	885.20 ± 30.04 ^bcd^	1029.20 ± 13.02 ^c^	1106.43 ± 10.42 ^c^	1155.17 ± 54.18 ^bc^	1310.83 ± 26.74 ^de^
	DS	855.20 ± 37.22 ^cd^	1095.1 ± 49.50 ^bc^	1148.67 ± 18.23 ^bc^	1193.70 ± 64.38 ^b^	1291.67 ± 9.00 ^e^
	F-value	Sc (F = 109.69 **) Re (F = 15.34 **) Sc × Re(F = 0.35)				
Shoot fresh weight (×10^−2^ g)	CK	21.77 ± 0.12 ^bc^	27.47 ± 0.09 ^d^	34.43 ± 0.39 ^d^	48.13 ± 0.45 ^c^	56.87 ± 1.02 ^c^
	N	25.00 ± 0.31 ^a^	29.20 ± 0.20 ^cd^	43.23 ± 1.28 ^b^	60.07 ± 0.82 ^a^	70.60 ± 2.03 ^a^
	A	22.53 ± 0.18 ^b^	34.80 ± 0.67 ^b^	47.97 ± 0.15 ^a^	53.77 ± 0.37 ^b^	62.20 ± 0.75 ^b^
	D	22.70 ± 0.17 ^b^	39.60 ± 0.32 ^a^	48.33 ± 1.12 ^a^	59.83 ± 1.13 ^a^	71.47 ± 1.98 ^a^
	S	19.70 ± 0.64 ^d^	21.20 ± 0.49 ^e^	32.77 ± 0.49 ^d^	44.10 ± 1.35 ^d^	52.50 ± 0.89 ^d^
	NS	24.70 ± 0.38 ^a^	27.27 ± 0.12 ^d^	39.13 ± 0.42 ^c^	53.07 ± 1.56 ^b^	54.23 ± 0.71 ^cd^
	AS	20.77 ± 0.24 ^d^	31.00 ± 0.70 ^c^	44.97 ± 1.78 ^b^	48.13 ± 0.57 ^c^	55.13 ± 0.32 ^cd^
	DS	20.37 ± 0.69 ^d^	31.13 ± 1.63 ^c^	39.90 ± 0.25 ^c^	46.53 ± 0.60 ^cd^	53.03 ± 0.19 ^d^
	F-value	Sc (F = 178.32 **) Re (F = 57.96 **) Sc × Re (F = 10.68 **)				
Shoot dry weight (×10^−2^ g)	CK	3.40 ± 0.12 ^ab^	4.67 ± 0.12 ^e^	7.07 ± 0.09 ^d^	9.17 ± 0.09 ^bc^	12.40 ± 0.15 ^bc^
	N	3.63 ± 0.32 ^a^	5.27 ± 0.176 ^cd^	8.93 ± 0.09 ^b^	11.40 ± 0.17 ^a^	13.37 ± 0.52 ^b^
	A	3.27 ± 0.09 ^ab^	6.37 ± 0.09 ^a^	9.97 ± 0.09 ^a^	11.80 ± 0.15 ^a^	14.60 ± 0.55 ^a^
	D	3.70 ± 0.06 ^a^	6.50 ± 0.06 ^a^	9.73 ± 0.38 ^a^	11.60 ± 0.06 ^a^	14.37 ± 0.09 ^a^
	S	3.07 ± 0.03 ^b^	3.8 ± 0.06 ^f^	6.03 ± 0.20 ^e^	8.00 ± 0.25 ^d^	10.07 ± 0.18 ^d^
	NS	3.63 ± 0.07 ^a^	4.97 ± 0.09 ^de^	7.4 ± 0.12 ^d^	8.77 ± 0.29 ^c^	11.43 ± 0.26 ^c^
	AS	3.17 ± 0.07 ^b^	5.73 ± 0.23 ^b^	8.27 ± 0.17 ^c^	9.40 ± 0.06 ^b^	12.00 ± 0.42 ^c^
	DS	3.10 ± 0.10 ^b^	5.53 ± 0.22 ^bc^	7.30 ± 0.17 ^d^	9.17 ± 0.12 ^bc^	11.60 ± 0.06 ^c^
	F-value	Sc (F = 149.76 **) Re (F = 41.27 **) Sc × Re (F = 1.65)				

## Data Availability

The raw data supporting the conclusions of this article will be made available by the authors on request.
